# BaM-seq and TBaM-seq, highly multiplexed and targeted RNA-seq protocols for rapid, low-cost library generation from bacterial samples

**DOI:** 10.1093/nargab/lqad017

**Published:** 2023-03-03

**Authors:** Grace E Johnson, Darren J Parker, Jean-Benoit Lalanne, Mirae L Parker, Gene-Wei Li

**Affiliations:** Department of Biology, Massachusetts Institute of Technology, Cambridge, MA, USA; Department of Biology, Massachusetts Institute of Technology, Cambridge, MA, USA; Department of Biology, Massachusetts Institute of Technology, Cambridge, MA, USA; Department of Physics, Massachusetts Institute of Technology, Cambridge, MA, USA; Department of Biology, Massachusetts Institute of Technology, Cambridge, MA, USA; Computational & Systems Biology Graduate Program, Massachusetts Institute of Technology, Cambridge, MA, USA; Department of Biology, Massachusetts Institute of Technology, Cambridge, MA, USA

## Abstract

The ability to profile transcriptomes and characterize global gene expression changes has been greatly enabled by the development of RNA sequencing technologies (RNA-seq). However, the process of generating sequencing-compatible cDNA libraries from RNA samples can be time-consuming and expensive, especially for bacterial mRNAs which lack poly(A)-tails that are often used to streamline this process for eukaryotic samples. Compared to the increasing throughput and decreasing cost of sequencing, library preparation has had limited advances. Here, we describe bacterial-multiplexed-seq (BaM-seq), an approach that enables simple barcoding of many bacterial RNA samples that decreases the time and cost of library preparation. We also present targeted-bacterial-multiplexed-seq (TBaM-seq) that allows for differential expression analysis of specific gene panels with over 100-fold enrichment in read coverage. In addition, we introduce the concept of transcriptome redistribution based on TBaM-seq that dramatically reduces the required sequencing depth while still allowing for quantification of both highly and lowly abundant transcripts. These methods accurately measure gene expression changes with high technical reproducibility and agreement with gold standard, lower throughput approaches. Together, use of these library preparation protocols allows for fast, affordable generation of sequencing libraries.

## INTRODUCTION

RNA sequencing (RNA-seq) is a powerful tool for quantitative measurement of the transcriptome and allows for global characterization of gene expression changes that aids in the discovery of novel gene regulatory mechanisms (1–3). However, the process of converting RNA samples into cDNA libraries compatible for sequencing on high throughput platforms is often labor-intensive and expensive (4–8), limiting the number of biological samples that can be analyzed in parallel. Recently, updated library preparation workflows have been developed that allow for early sample barcoding and pooling to streamline this process. However, challenges still remain for processing bacterial samples, as existing protocols largely rely on barcoding via oligo(dT) primers (9, 10) that cannot capture bacterial mRNAs. Alternative protocols suitable for bacterial mRNAs utilize random hexamer priming (11) or introduce additional ligation steps (12), which can lead to potential bias in the recovered libraries (13, 14) and, when ligation is required, increase the final cost per sample.

Further increasing the cost of RNA-seq experiments is the skewed composition of most transcriptomes in which a small number of highly expressed transcripts represent the majority of RNA molecules. In bacterial samples, the top 1% most highly expressed genes account for 30% of all mRNA reads, whereas only 1% of mRNA reads map to the bottom 50% of genes (8). Thus, quantification of more lowly expressed RNAs requires redundant counting of abundant molecules, drastically increasing the total number of reads required to profile a given sample. Approaches now exist to enrich libraries for specific targets, biasing towards genes of interest and decreasing required sequencing depth and cost (5, 15–17). Such enrichment is frequently achieved through capture with hybridization probes (CaptureSeq) (18–21), which is able to accurately quantify the expression of all but the most highly abundant RNAs (22). However, hybridization adds several additional steps to protocols and is typically completed over many days (18).

Here, we describe an alternative to existing multiplexed RNA-seq protocols, bacterial-multiplexed-seq (BaM-seq). BaM-seq rapidly converts RNA into barcoded cDNA in a single tube, enabling early pooling of samples that streamlines downstream processing and increases throughput. Further, we describe a target-enrichment strategy, targeted-bacterial-multiplexed-seq (TBaM-seq), that can be applied to pooled cDNA samples, involving a second-strand synthesis reaction with specific priming for fast and highly customizable target-enrichment. We measure robust target-enrichment for non-rRNA depleted samples, obviating the need for time consuming and costly rRNA removal. Lastly, we demonstrate how TBaM-seq can be used to redistribute reads between transcripts to measure expression changes of both highly and lowly expressed genes with minimal sequencing reads. We find that these methods allow for highly reproducible expression quantification that agrees well with previously established protocols. Our approach uses inexpensive reagents and represents a strategy for cheaper and faster library generation that can be performed in most laboratories.

## MATERIALS AND METHODS

### Strains

*Escherichia coli* MG1655 K12 and *Bacillus subtilis* 168 (*trpC2)* were used as wildtype strains to test reproducibility across barcodes for the multiplex and targeted workflow, respectively (Figures 2A, B and 4A, B). *E. coli* Δ*fis* and Δ*ahcP* strains (Figure 2C), produced as part of the Keio collection (23), were obtained from the Coli Genetic Stock Center (CGSC) at Yale University. *B. subtilis* Δ*rho* strain (Figure 4C), produced by Koo *et al.* (24), was obtained from the *Bacillus* Genetic Stock Center (BGSC) at The Ohio State University. The PspankHY-*lacZ B. subtilis* strain (Figure 4D) was described previously (25). Paired Rend-seq and multiplex RNA-seq was performed on *B. subtilis* strain GLB455, which contains an inducible GFP-RFP fusion at *amyE* (26).

### Cell growth and collection

All cells were grown in LB media. To collect *E. coli* cultures, overnight cultures were started from single colonies, and back-diluted >400-fold into fresh LB. At an OD_600_ = 0.3, 5 ml of cells were mixed with 5-ml ice cold methanol, spun for 10 min, decanted, and stored at –80°C. To collect *B. subtilis* cultures, single colonies were picked into 10 ml LB and grown for 2–3 h. Cultures were back-diluted to an OD_600_ = 0.0002 in 15 ml fresh LB. At an OD_600_ = 0.2, 7 ml of cells were collected into 7-ml ice cold methanol, spun for 10 min at 4°C, decanted, and stored at –80°C. For the paired Rend-seq and multiple RNA-seq experiment (Figure 2D), cultures were collected as for other *B. subtilis* strains, with starter cultures back diluted into LB containing 0% xylose and 0 μM IPTG (sample A), 0.2% xylose and 100 μM IPTG (sample B), or 0.5% xylose and 1000 μM IPTG (sample C). For IPTG titration (Figure 4D), cultures were collected as for other *B. subtilis* strains, with starter cultures back-diluted into 15 ml LB containing either 0, 10, 20, 30 or 100 μM IPTG.

### RNA extraction and rRNA removal

RNA was extracted and gDNA depleted using RNeasy Plus mini kit (Qiagen) following manufacturer's instructions. For RNA samples prepared by Rend-seq or the BaM-seq protocol, rRNA was subsequently depleted using MICROBExpress Bacterial mRNA enrichment kit (Invitrogen) as follows. 20 μg RNA, in a max volume of 30 μl, was added to 0.4 ml binding buffer. 8 μl capture oligo mix was added and the reaction incubated at 70°C for 10 min and 37°C for 15 min. Oligo magbeads were prepared by washing beads with 100 μl water, followed by 100 μl binding buffer. Beads were resuspended in 100 μl binding buffer and heated to 37°C. 100 μl oligo magbeads were added to the RNA/capture oligo mix and incubated at 37°C for 15 min and supernatant recovered into a fresh tube. Beads were washed with 150 μl 37°C wash solution and supernatant recovered into the same tube. RNA was then precipitated and resuspended in 40 μl 10 mM Tris 7.0. For experiments in which technical replicates were used, samples were split following RNA extraction or rRNA removal.

### RNA-seq library preparation

BaM-seq libraries were generated as detailed in the protocol found in the supplemental method. Briefly, 250 ng rRNA depleted RNA was brought to 10 μl in H_2_O and fragmented at 95°C for 1 min 45 s with 1 μl 10× fragmentation reagent (Invitrogen). The fragmentation reaction was stopped with 1.1 μl Stop solution and cleaned up with Zymo Oligo Clean and Concentrator Columns following manufacturers’ instructions and eluted in 16 μl H_2_O. To dephosphorylate the RNA, 2 μl 10× PNK Buffer (NEB), 0.25 μl SUPERase*In (Invitrogen), 1.25 μl DEPC H_2_O, and 0.5 μl PNK enzyme (NEB) were added to each sample and samples incubated at 37°C for 60 min and 75°C for 10 min. Polyadenylation was performed by adding 10 μl PolyA Master Mix (NEB) containing 3 μl 500 mM KCl, 3 μl 10 mM ATP, 2 μl 5× FS Buffer, 0.25 μl SUPERase*In, 1.25 μl H_2_O, and 0.5 μl *E. coli* PolyA polymerase (NEB) to samples and incubating at 37°C for 30 min and 75°C for 10 min. Subsequently, 1 μl of 25 μM RT Barcoding primer was added to each sample and incubated at 65°C for 5 min, then returned to ice. 3 μl 0.1M DTT, 2 μl 10 mM dNTP mix, 2 μl 5× FS Buffer (Invitrogen), 1.25 μl DEPC H_2_O, 0.25 μl SUPERase*In, and 0.5 μl SSIII RT Enzyme (Invitrogen) was added and the reaction incubated at 50°C for 60 min and 75°C for 10 min. All samples were pooled into a single tube and mixed thoroughly, and RNA degraded by adding 0.1 volume of 1M NaOH and incubating at 95°C for 15 min. 180 μl of the pooled sample was run on a 10% TBU gel (Invitrogen) and material between 100–120 nt cut and extracted. DNA was precipitated and dissolved in 20 μl 10 mM Tris 8. 5 μl adapter was ligated to 10 μl of cDNA in a reaction containing 3 μl DEPC H_2_O, 5 μl 10× T4 DNA ligase buffer, 5 μl 5M Betaine, 20 μl PEG 8000, and 2 μl T4 DNA ligase (NEB) that was incubated at 16°C for 10 h. The enzyme was denatured by heating the reaction to 75°C for 10 min and the reaction subsequently cleaned with a Zymo oligo clean and concentrator column following the manufacturers’ instructions. The reaction was run on a 10% TBU gel for 1 h and 45 min and the band between 135 and 155 nt cut, extracted, precipitated, and cDNA resuspended in 20 μl 10 mM Tris8. A PCR mastermix containing 5 μl ligated DNA, 6 μl 10 μM oDP161, 6 μl 10 μM oDP128, 6 μl 10 mM dNTP mix, 24 μl 10× Q5 buffer (NEB), 60 μl water, and 2 μl Q5 polymerase (NEB) was prepared and aliquoted into five 20 μl reactions that were run at each of 5 cycles: 6, 8, 10, 12 and 14 cycles. Samples were run on an 8% TBE gel and the final product recovered. All oligo sequences are included in the supplementary method.

TBaM-seq libraries were generated as detailed in the protocol found in the supplementary methods. Briefly, 250 ng of total, non-rRNA removed RNA was fragmented for 30 s at 95°C as described above. Samples were dephosphorylated, polyadenylated, and reverse transcribed as described above. 240 μl pooled RT reaction was run over two 10% TBU gels and product between 115 and 135 nt size selected. The recovered RT product was resuspended in 40 μl 10 mM Tris 8 and 32 μl 5x Phu HF Buffer, 3.2 μl 10 mM dNTPs, 1 pmol of each primer, 4.8 μl DMSO, and 1.6 μl Phu Polymerase (NEB) added and brought to a total volume of 160 μl with water. The reaction was incubated at 98°C for 30 s, 58°C for 15 s, and 72°C for 30 s and subsequently run on a 10% TBU gel to size select products between 145 and 165 nt. The product was resuspended in 20 μl 10 mM Tris pH 8 and final PCR performed as described above with 16, 18, 20, 22 and 24 cycles.

REND-seq was performed as described previously (8).

All samples were sequenced on either a HiSeq2000 or NextSeq500. BaM-seq samples were sequenced at a depth of 10–50 million reads per sample (Figure 2A samples, 10 million reads, Figure 2D samples, 50 million reads, Figure 4D, 15 million reads). A summary of TBaM-seq samples, including sequencing depth, is included as supplementary data.

### RNA-seq data analysis

RNA sequencing reads were processed by removing poly(A) tails with cutadapt (options -a AAAAAAAAAAAAAAAAAAAA;min_overlap = 10) and mapped using Bowtie (27) (options -v 2 -k 1 –best) to the NC_000964.3 reference genome for *B. subtilis* or NC_000913.3 for *E. coli*. Total reads per gene for multiplex and Rend-seq datasets were calculated from 5’ mapped reads excluding the first and last 30 nt of the CDS.

Gene expression for Rend-seq and TBaM-seq datasets was calculated as reads per million (rpm) for genes with more than 100 mapped reads. To compare correlation between technical replicates (as in Figures 2A and 4A), the *r* value for all pairwise samples was calculated from log_10_-transformed data.

For TBaM-seq samples, bowtie output files were first adjusted by adding the read length to mapped position for reverse strand mapping reads. Primer sequences were also mapped to the same genome, and bowtie output files similarly adjusted. Reads were then assigned to specific primers by matching location of mapped reads to priming locations. Expression measured by each primer is reported as reads corresponding to that primer location per million primer reads mapped (rpm). To measure the expression of a gene captured by multiple primers, the median primer rpm was used. To identify non-specific priming events, fastq files of unmapped reads were split into two separate files, one with the first 20 nt of the read and one with the remainder of the read. These files were then remapped to the genome with bowtie as above. Reads where the first 20 nt mapped to a location of a priming site were characterized as non-specific priming events. The location of mispriming was subsequently identified using the mapping of the remainder of the read.

### Calculating barcode switching frequency

In the knockout experiment, barcode switching frequency was calculated as the fraction of reads mapped to a CDS or TBaM-seq primer in the knockout versus WT strain divided by the total number of target gene containing samples in the pool.

For the mixed species barcoding experiment, we analyzed an unpublished 95-million-read BaM-seq run which included both a barcoded *B. subtills* (WT 168) sample (∼20 million reads) and several barcoded *E. coli* (MG1655 with an overexpression plasmid) samples (∼70 million reads across three barcodes). Barcode switching frequency was calculated as the number of *B. subtills* barcoded reads mapping to the *E. coli* genome (157509 reads) divided by the total number of uniquely mapped *E. coli* reads in the pool (11936187 reads), or 1.3%. The library also contained a fifth sample that was intentionally mixed (both *B. subtilis* and *E. coli* RNA) that accounts for a small fraction of total reads. We have omitted this sample from our analysis, and thus the reported barcode switching frequency represents an upper bound on the actual frequency.

### Targeted primer design

Primers were designed as forward primers off coding strand sequences using Primer3 (28, 29) with the following parameters: optimal size = 20 nt, maximum size = 22 nt, minimum tm = 53°C, optimal tm = 55°C, max tm = 56°C. Input sequences and parameters were specified using the template provided with Primer3. For all returned primers, all 8-nt fragments within the primer were aligned to *B. subtilis rrn* operons using bowtie (27) (options -v 0 -k 1 –best), and any primer with an alignment was removed. From the remaining primers, a final primer set was selected such that the 5’ end of any given primer annealed at least 20 nt downstream the 3’ end of the closest upstream primer. The common adapter sequence CTTTCCCTACACGACGCTCTTCCGATCT was appended to the 5’ end of all primers. Targeted primer sequences are included as supplementary data.

### Demonstration of redistribution

As a theoretical demonstration of the benefit of read distribution through use of multiple second strand synthesis reactions, we considered the top 1000 most highly expressed genes in the *B. subtilis* transcriptome as determined using a high-depth RNA-sequencing dataset (8). Genes were ordered by expression, with gene one being the most abundant gene, and gene 1000 being the 1000th most abundant gene. The number of reads required to sequence each of these genes with at least 100 reads per gene using non-targeted approaches was calculated by dividing 100 by the number of reads mapped to the 1000th gene in (8) and multiplying the sum of reads mapped to the top 1000 genes by the resulting number. To calculate the number of reads required to obtain the same information using TBaM-seq, the top 1000 genes were divided into five equal groups each containing 200 genes (group 1 contains genes 1–199, group 2 contains genes 200–399, etc.). The number of reads required to sequence each gene with at least 100 reads was then calculated separately for each of the five groups and summed. Similar to above, 100 was divided by the number of reads mapped to the least abundant gene in the group and the sum of all reads in the group multiplied by the resulting number to get the number of reads required to sequence that group.

## RESULTS

### An RNA-seq protocol with early barcoding

**Figure 1.**
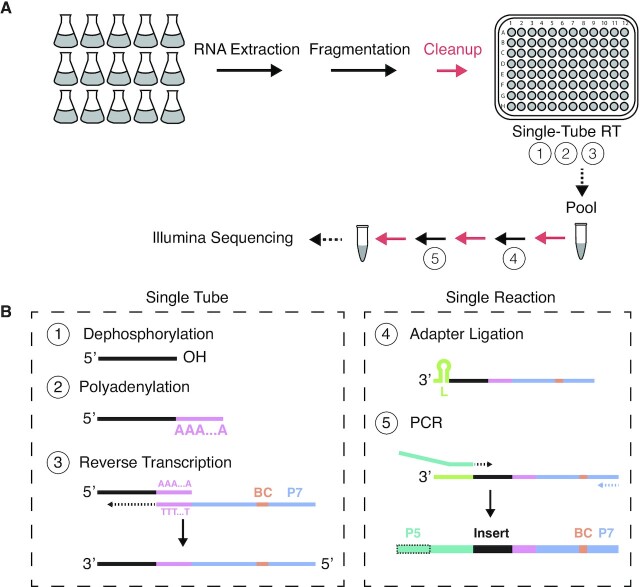
BaM-seq library preparation workflow. (**A**) Fragmented RNA can be rapidly converted into barcoded cDNA libraries via a single-tube RT reaction with no intervening clean-up steps. RT products can be subsequently pooled, and downstream processing steps are performed as a single sample. Red arrows indicate clean-up steps. (**B**) The single-tube RT reaction, steps 1–3, involves dephosphorylation of RNA by T4 polynucleotide kinase, polyadenylation of 3’ ends by *E. coli* Poly(A) Polymerase, and reverse transcription by SSIII using barcoded oligo(dT) primers. Following pooling, an adapter is ligated to the 3’ end of cDNA molecule (step 4), and libraries PCR amplified (step 5).

To streamline the process of generating sequencing-compatible cDNA libraries, we developed BaM-seq, a protocol that allows for early barcoding and pooling of samples (Figure 1). Such early pooling increases the throughput of RNA-seq experiments by allowing multiple samples to be processed simultaneously in a single reaction. Barcoding of eukaryotic samples can be obtained by priming mRNA poly(A)-tails with barcoded oligo(dT) primers (9,10). However, this approach cannot be applied directly to bacterial mRNAs which lack such poly(A)-tails. Barcoding of bacterial RNA samples may utilizes adapter ligation, but preadenylated RNA or DNA adapters are expensive to generate (12). To barcode bacterial RNA samples, we took advantage of the commercially available *E. coli* Poly(A) Polymerase that can efficiently adenylate RNA 3’ ends *in vitro* independent of terminal nucleotide identity, thus allowing us to use barcoded oligo(dT) primers to mark the cDNAs and subsequently pool them, analogous to (30). Prior to polyadenylation, RNA samples are fragmented to increase the number of 3’ ends amendable to polyadenylation and enable more even coverage across the length of transcripts.

We developed a simple protocol to perform sequential reactions in a single tube without intervening clean-up steps to convert fragmented RNA into barcoded cDNA. This protocol involves dephosphorylation of RNA 3’ ends by T4 Polynucleotide Kinase, poly(A) tailing by *E. coli* Poly(A) Polymerase and reverse transcription (RT) by Superscript III (SSIII) with barcoded oligo(dT) RT primers. Following RT, barcoded cDNA samples can be pooled, and downstream steps of size selection, 3’ adapter ligation, and PCR amplification can be performed on this single pooled sample (Figure 1). Together, our protocol allows for highly multiplexed sequencing library generation whereby many samples can be processed together.

### Validation of BaM-seq protocol

**Figure 2.**
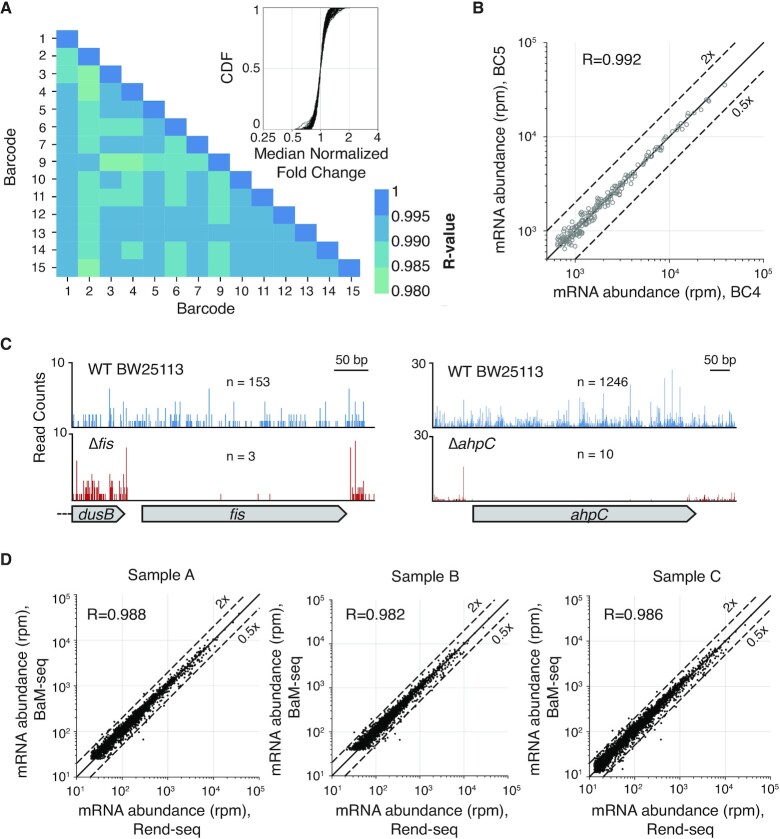
Validation of BaM-seq protocol. (**A**) Pearson correlation of log_10_-transformed rpm values for genes with at least 100 mapped reads (212 genes) between 14 technical replicates. Minimum *R*-value = 0.983. The inset shows the cumulative distribution of median-normalized fold-changes for all pairs of genes between all pairwise combinations of replicate samples. (**B**) Representative example of rpm correlation between two replicates as in (A). Genes with more than 100 mapped reads in both samples are plotted. (**C**) 5’ mapped reads across *fis* and *ahpC* genomic loci in WT (153 and 1246 mapped reads, respectively), and Δ*fis* (three mapped reads) and Δ*ahpC* (10 mapped reads) *E. coli* strains. (**D**) Relative expression of genes as measured from three split RNA samples processed with BaM-seq or Rend-seq. Rpm was plotted for all genes with >100 reads in both samples.

We next tested the performance of our BaM-seq protocol. We first demonstrated that the gene expression profiles of differentially barcoded samples were highly correlated. To show this, we split an RNA sample from *E. coli* BW25113 into 14 independent replicates and barcoded each with a unique index such that they could be pooled, processed, and sequenced together. We measured a high degree of correlation of relative gene expression levels between samples (Figure 2A, B, minimum *R* = 0.983, median SD for log_2_ fold-change = 0.176), demonstrating the technical reproducibility of our protocol. The correlation between barcodes is higher than that observed with RNAtag-seq (12), an alternative method for early multiplexing of bacterial mRNA samples by ligating to barcoded adapters. In addition, as all barcodes tested here demonstrated strong correlation, this approach does not require pre-validation of barcodes, unlike is necessary for barcoded ligation adapters, that can add additional time and cost to an experiment (12).

One potential drawback of early barcoding is barcode-switching during downstream reactions, most commonly during PCR amplification (31,32), whereby a barcode from one sample becomes mis-associated with an insert from a different sample. A high frequency of barcode-switching can be especially problematic when many samples are pooled as it can mask gene expression changes that occur in only one or a small fraction of samples. To test the prevalence of barcode-switching in our protocol, we compared the expression of *fis* and *ahpC* between WT and Δ*fis* and Δ*ahpC* strains, respectively, that were prepared and sequenced in a pool with 14 other samples with WT-levels of *fis* and *ahpC* (33). This experiment represents a stringent test of barcode-switching, as the knockout samples completely lack a gene that is present in all other samples in the pool. Reads were strongly depleted in the coding sequences (CDSs) of deleted genes in knockout strains, with an estimated barcode-switching frequency of less than 0.15% between two samples in the same pool (Figure 2C, see Materials and Methods). In addition, we also assessed barcode-switching in pools containing RNA from both *E. coli* and *B. subtilis* by calculating the occurrence of *E. coli* mapping reads with a barcode corresponding to a *B. subtilis* RNA sample. With this approach, we estimate barcode-switching occurs for at most 1.3% of molecules (see Materials and Methods). These results indicated minimal barcode crosstalk by our approach.

Lastly, we confirmed that our approach is able to accurately capture relative transcript abundances as measured by established RNA-seq approaches. We generated sequencing libraries from three *B. subtilis* RNA samples by both BaM-seq and End-enriched RNA-seq (Rend-seq) (8). Rend-seq is a lower throughput protocol that allows for both 5’ and 3’ end mapping as well as gene expression quantification, and measures RNA levels consistent with other gene expression datasets (R^2^ = 0.8, Rend-seq v. microarray) (8). For all samples, there was strong correlation between the gene expression profiles measured by the two approaches (Figure 2D, minimum *R* = 0.982, median SD of log_2_ fold-changes = 0.326). BaM-seq also demonstrated the same sensitivity as Rend-seq, with both approaches detecting the same fraction of genes at a given sequencing depth (Supplementary Figure S2). With a sequencing depth of 50 million reads (5 million mRNA mapping reads), BaM-seq can reliably measure the expression of genes expressed at a level of 2 transcripts per million or higher. Thus, our BaM-seq protocol retains the quantitative information about the transcriptome captured by lower throughput methods, even when many samples are pooled.

### Target-enrichment following RT using TBaM-seq

**Figure 3.**
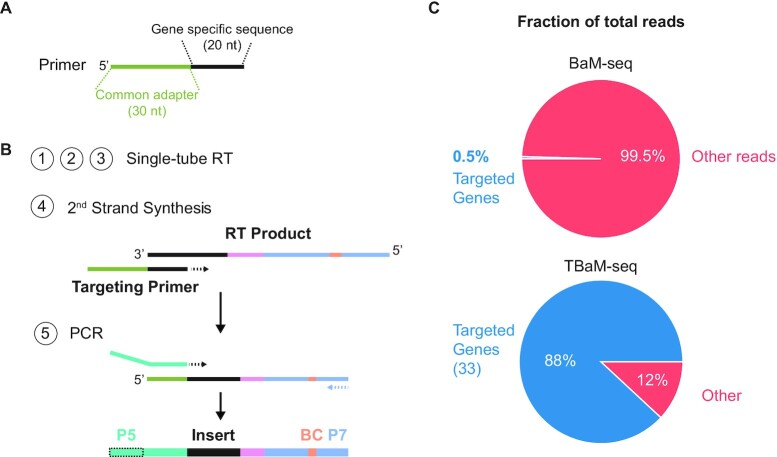
Overview of TBaM-seq protocol. (**A**) Primers for target-enrichment, each containing a 30-nt common adapter and 20-nt gene specific homology region. (**B**) Following single-tube RT, cDNA libraries can be enriched for transcripts of interest via a second-strand synthesis reaction with gene-specific primers as shown in (A) and subsequently PCR-amplified from the common adapter. (**C**) The percentage of reads mapping to 82 targeted genes in libraries prepared with either BaM-seq or TBaM-seq.

To further increase the number of samples that can be processed and sequenced together with a finite number of reads, we developed a target-enrichment protocol that can be applied following RT and pooling, TBaM-seq. Our approach utilizes target-specific primers, each containing a common adapter and 20 nts of complementarity to a particular RT product (Figure 3A) that can template synthesis of a second DNA strand. This second-strand synthesis reaction replaces the adapter ligation step of our multiplexed protocol, with downstream PCR amplification primed from the common adapter such that only targeted products are retained (Figure 3B). Given the ease and relatively low cost of custom DNA oligo synthesis, customized primer pools can be readily designed to enrich for subsets of transcripts relevant to the scope of an experiment.

To test our ability to enrich for genes of interest while minimizing off-target capture, we designed a pool of 162 second-strand primers targeting 82 *B. subtilis* genes whose expression spans over 2 orders of magnitude. We designed primers such that no 8-nt stretch shared homology with any rRNA, as primers containing homology to rRNA operons, regardless of where the homology was located within the primer, readily mis-primed and led to final libraries that were comprised almost exclusively of such molecules (Fig. S1B). With this design approach, we were able to achieve libraries with ∼90% of reads derived from specific priming events (Fig. S1A). The remaining nonspecific priming events can be filtered out bioinformatically (Fig. S1A). This represents an over 100-fold enrichment in reads from target genes as compared to standard multiplexed libraries, where only 0.5% of reads map to targeted genes (Figure 3C). It is worth noting that this enrichment is underestimated, as multiplex libraries are generated from RNAs that are depleted for abundant rRNA (34), whereas targeted libraries are generated directly from total RNA without rRNA depletion. As such, this method is able to effectively capture transcripts of interest with little off-target priming, thereby reducing required sequencing depth, eliminating the need for rRNA removal, and decreasing experimental cost.

### Validation of TBaM-seq protocol

**Figure 4.**
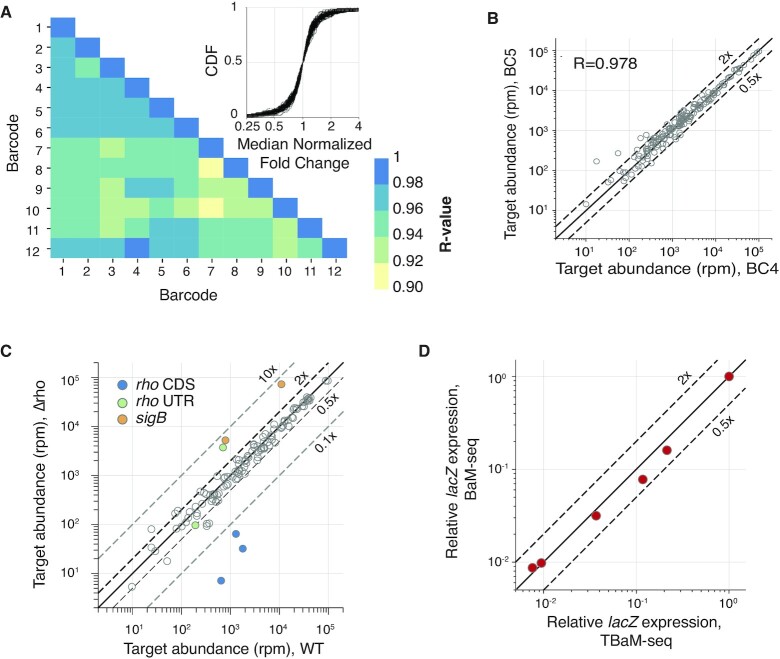
Validation of TBaM-seq protocol. (**A**) Pearson correlation between replicates of log_10_-transformed reads per second-strand primer. (**B**) Representative example of correlation across second-strand primers between replicates. A total of 162 second-strand primers targeting 82 genes were included in this pool. The second-strand primers that show >2-fold difference in rpm between these two samples target the genes *polA* (0.10× between sample 4 and 5), *spo0E* (0.20), *lysC* (0.28), and *glnR* (0.46). *polA*, *spo0E*, and *lysC* are the three lowest expressed genes targeted by this second-strand primer pool (as measured by non-targeted BaM-seq). (**C**) Reads per million primer-mapping reads for each second-strand primer between WT and Δ*rho* strains. Primers targeting *rho* CDS and UTR, as well as those targeting *sigB* are highlighted. (**D**) *lacZ* expression relative to sample with highest *lacZ* induction. For BaM-seq samples, *lacZ* expression is calculated as rpkm. For TBaM-seq samples, *lacZ* expression is calculated from median normalized reads across 12 *lacZ*-targeting primers.

We tested consistency between barcodes following target capture by splitting an RNA sample from WT 168 *B. subtilis* into 12 reactions that were barcoded, pooled, and subject to our targeting protocol using the pool of 162 second-strand primers. Following sequencing, we observed good correlation of reads mapping to each primer between samples (Figure 4A, B, minimum *R* = 0.918, median SD for log_2_ fold-changes = 0.793). The variability that did exist across samples largely came from primers targeting regions of the most lowly expressed genes we aimed to capture (Figure 4B). Detection of these lowly abundant fragments is not limited by sequencing depth in our experiments, but likely represents a lower bound of target capture by our approach.

We next evaluated the potential of barcode-switching that could additionally occur during the second-strand synthesis reaction. To measure the extent of barcode-switching, we prepared a pooled library containing five WT samples and one Δ*rho* sample. In the Δ*rho* sample, *rho* CDS targeting primers measured *rho* expression 1–5% that of WT levels, indicating a crosstalk rate of 0.2–1% between two samples in the same pool (Figure 4C). We hypothesize that barcode-switching largely results from priming of second-strand products by residual RT primer during second-strand synthesis. Indeed, when we increase the number of second-strand synthesis cycles from one to ten, the barcode-switching rate between two samples increases (4%) (Supplementary Figure S3A).

Targeted primers can also be designed to provide position-specific information. For example, using second-strand primers targeted to the 5’ UTR of *rho*, we can measure increases in *rho* 5’ UTR expression upon *rho* deletion, consistent with the negative autoregulation of this gene (35) (Figure 4C). We can also measure other gene expression changes known to be associated with *rho* deletion (8), such as upregulation of *sigB*.

To further demonstrate that TBaM-seq could measure gene expression changes, we compared the relative gene expression of an exogenous copy of IPTG-inducible *lacZ* in a *B. subtilis* strain measured by BaM-seq or TBaM-seq. The relative expression across different IPTG concentrations measured from 12 *lacZ*-targeting second-strand primers agreed well with that calculated from whole-transcriptome BaM-seq (Figure 4D). We were also able to measure gene expression changes with single second-strand primers at all but the lowest expression levels of *lacZ*. Expression changes measured by single primers were within ∼2-fold of those measured without targeting, except for strains grown without IPTG, where signal measured between primers differed >10-fold (Supplementary Figure S3B). The leaky expression of *lacZ* without IPTG is lower than that of 1200 endogenous *B. subtilis* genes. Thus, while single primers are likely sufficient to quantify the expression of many abundant transcripts, use of additional primers is recommended for capturing lowly expressed genes. Lastly, our targeted approach was able to estimate relative gene expression levels between endogenous transcripts targeted in our experiment, with increased agreement with non-targeted approaches if expression was measured with multiple second-strand primers (Supplementary Figure S3C, *R* = 0.77 between median primer reads from TBaM-seq and BaM-seq, *R* = 0.99 for genes with >8 primers). Together, these results demonstrate that TBaM-seq provides robust measurements for differential expression of the same gene across different conditions. For comparing relative expression between different genes in the same condition, multiple primers per gene should be used.

### Redistribution of reads using TBaM-seq

We reasoned that we could further decrease the required sequencing depth by performing separate target-enrichment reactions for pools of genes with different expression levels. By capturing highly abundant transcripts in a separate reaction, it should be possible to measure the expression of these genes without redundantly counting them at the expense of more lowly abundant transcripts. To demonstrate this, we performed two second-strand synthesis reactions on the same pool of WT *B. subtilis* replicates with two distinct sets of primers (pool 1 and pool 2 primers). Pool 1 contained 112 primers targeting 32 genes, and pool 2 contained 50 primers targeting 50 of the most highly expressed genes. In non-targeted BaM-seq experiments, pool 1 genes receive only 20% of reads as compared to pool 2 genes (Supplementary Figure S4B). However, following two separate enrichment reactions and subsequent re-pooling and sequencing, we were able to enrich for pool 1 genes, such that pool 1 primers instead received 1500% the number of reads as pool 2 primers (Supplementary Figure S4A). By altering the pooling ratio between two or more second-strand synthesis reactions, the number of reads dedicated to any given pool can be tailored to the specific experiment. Use of multiple primer pools can successfully redistribute reads to more lowly expressed transcripts, decreasing required sequencing depth and cost.

## DISCUSSION

We have developed a highly multiplexed RNA-seq protocol with an optional target-enrichment step that allows for high-throughput processing of many RNA samples at once. For both approaches, we have demonstrated that they produce consistent results across technical replicates, exhibit low levels of crosstalk between samples, and retain quantitative information measured by lower throughput methods. Our BaM-seq workflow rapidly converts fragmented RNA into barcoded cDNA in a single tube without requiring intervening clean-up steps. In addition to simplifying sample processing, this approach drastically reduces the cost of library preparation. Although this does not include the cost of rRNA removal, which must be performed prior to downstream processing, when paired with ‘do-it-yourself’ rRNA removal methods (36), our multiplex strategy represents a highly cost-effective approach for generating sequencing libraries.

The TBaM-seq protocol is able to enrich for transcripts of interest by over 100-fold while still accurately measuring their expression. Target selection is achieved through use of short 50-nt oligos comprised of a common handle and target-specific sequence. The ease of synthesizing short custom oligos means that primer pools can be readily obtained to tailor target-enrichment for specific experiments. There are several important factors to consider when designing such second-strand primers for a given application. First, second-strand primers should avoid homology to highly abundant transcripts to avoid off-target capture. In the bacterial RNA samples used here, rRNA comprises the vast majority of RNA molecules, and primers were thus designed specifically without rRNA homology. However, in different contexts, primer design may need to consider other abundant RNAs. Second, the number of second-strand primers per gene can be varied depending on the information desired. As primer efficiency for most primers is consistent between samples, our targeted approach can readily measure many gene expression differences between samples with just a single primer. However, as primer efficiency can vary from primer to primer, more primers should be used per gene to measure quantitative difference between expression of genes in a single sample, in order to average out inter-primer heterogeneity, or to accurately quantify the expression of lowly abundant genes.

Our targeted approach is accurate and reproducible for all but lowly expressed genes. Variability in measuring these low-abundance transcripts by our method may result from stochasticity in priming. Noise could therefore be reduced by increasing signal through the use of additional primers or by increasing the amount of input material. Although individual primers targeting *lacZ* yielded signals spanning an over 10-fold range in strains where *lacZ* expression was not induced, taking the median across these primers allowed for more accurate expression quantification that agreed with a non-targeted approach. Although we have demonstrated that rRNA removal is not required for quantification of most transcripts, depleting samples for such abundant RNAs would likely lower this limit of detection by both increasing the concentration of mRNA targets of interest as well as reducing non-specific interactions in the second-strand synthesis reaction. rRNA depletion may therefore facilitate the capture of rare transcripts.

We have also demonstrated how TBaM-seq can be used to redistribute reads and thereby decrease the sequencing depth required to measure the expression of lowly abundant transcripts. With traditional RNA-seq approaches, 20 million mRNA reads are required to cover the top 1000 genes in the *B. subtilis* transcriptome with at least 100 reads per gene. By contrast, capturing these transcripts in five separate second-strand synthesis reactions, each targeting 200 similarly expressed genes, can in principle reduce the required number of reads over 20-fold to 700,000 (see Materials and Methods). The reduction in required reads is even more dramatic when considering measurement of a set of genes comprising > 99% of the *B. subtilis* genome, decreasing the required depth 1000-fold from 4 billion reads to 4 million (8). Ability to capture the lowest abundance transcripts may be achievable by increasing the input material or using more primers per gene, as described above.

Lastly, whereas our target-enrichment protocol was developed to be compatible with our BaM workflow, it can in theory be applied to any cDNA library and therefore represents a highly adaptable tool. This approach may be particularly useful for applications such as measuring pathogen mRNAs in host-pathogen pools, or capturing species-specific transcripts from multi-microbial communities. Additionally, while both methods were designed and tested to streamline processing of bacterial samples, they could also serve as useful tools for sequencing eukaryotic samples, particularly non-adenylated and low abundance RNAs.

The protocols described here represent an alternative RNA-seq approach that allows for highly multiplexed library generation. Our early barcoding provides the ability to easily scale up experiments with little increase in time or cost required to generate sequencing-ready libraries. Addition of a targeting step further decreases the cost of downstream sequencing by decreasing the required sequencing depth. Together, these methods allow for accurate and easy measurements of bacterial transcriptomes.

## DATA AVAILABILITY

Published sequencing datasets analyzed in this paper are available from the Gene Expression Omnibus repository with accession numbers GSE162169 (TBaM-seq v. Rend-seq comparison), GSE129161 (TBaM-seq barcode switching) and GSE95211 (calculation of read reduction through tiering). All sequencing data generated as part of this study can also be downloaded with accession number GSE206425.

## Supplementary Material

lqad017_Supplemental_Files

## Notes

Present address: Grace E. Johnson, Department of Molecular Biology, Princeton University, Princeton, NJ, USA.

Present address: Darren J. Parker, Evozyne Inc., Chicago, IL, USA.

Present address: Jean-Benoit Lalanne, Department of Genome Sciences, University of Washington, Seattle, WA, USA.
