# Supplementary material for: BaM-seq and TBaM-seq, highly multiplexed and targeted RNA-seq protocols for rapid, low-cost library generation from bacterial samples
Source: NAR Genom Bioinform. 2023 Mar 3;5(1):lqad017. doi: 10.1093/nargab/lqad017 (PMC9985320; doi:10.1093/nargab/lqad017)

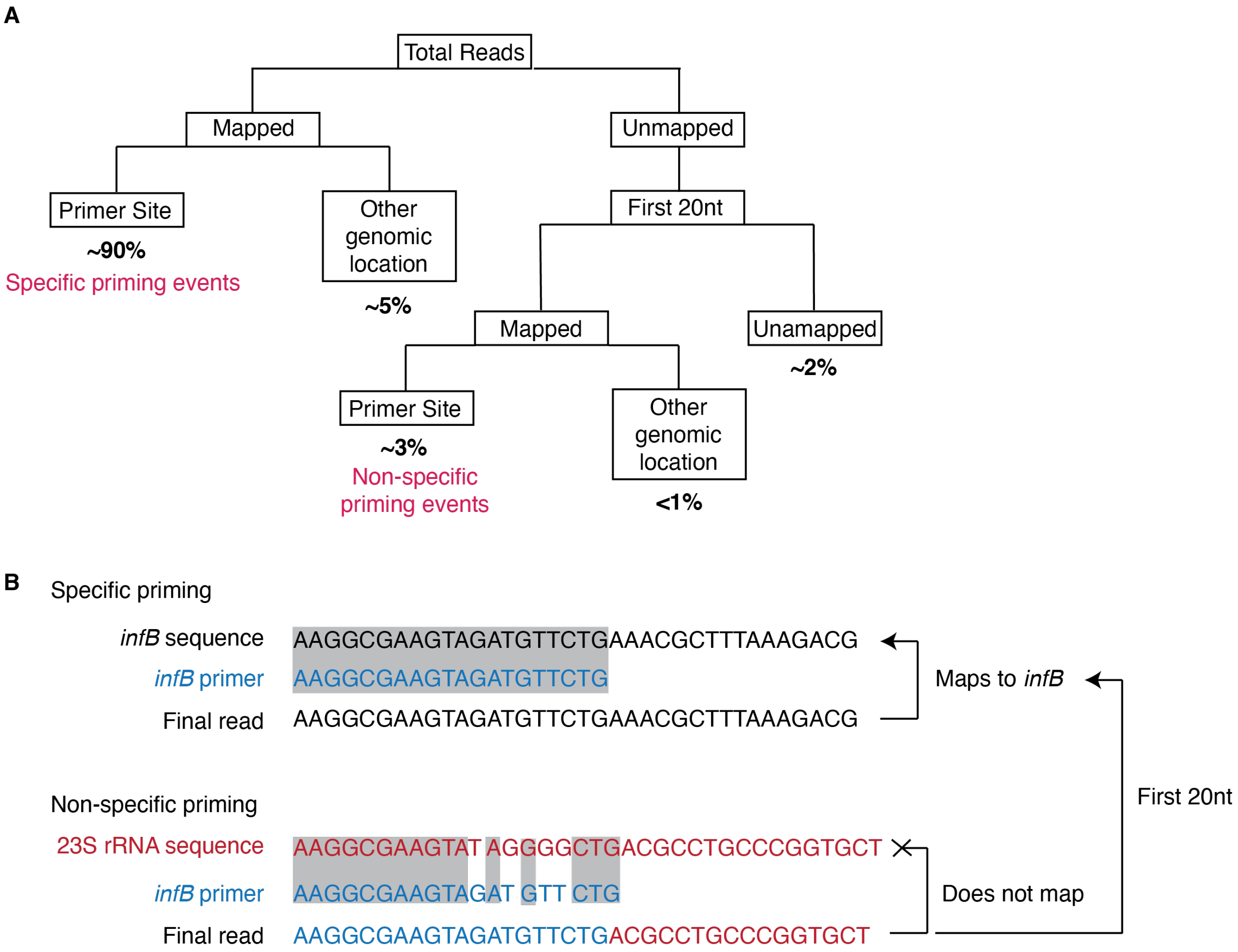

Figure S1. Identifying specific and non-specific priming events following sequencing of TBaM-seq samples. (A) Schematic of read mapping to identify specific and non-specific priming events. Reads that map to a specific primer site are characterized as specific priming events (~90% of reads). The first 20 nt of unmapped reads are re-mapped and those that map to a primer site are characterized as non-specific priming events (~3% of reads). Remaining reads map to other genomic locations or do not map to the *B. subtilis* genome. (B) Example of a non-specific priming event resulting from a primer with shared sequence identity to 23S rRNA. Complementarity between the 5' end of the primer and cDNA molecules generated from the 23S rRNA is sufficient to prime second-strand synthesis at this off target site. Resulting reads are comprised of a chimera of the primer sequence and region downstream the off-target priming site.

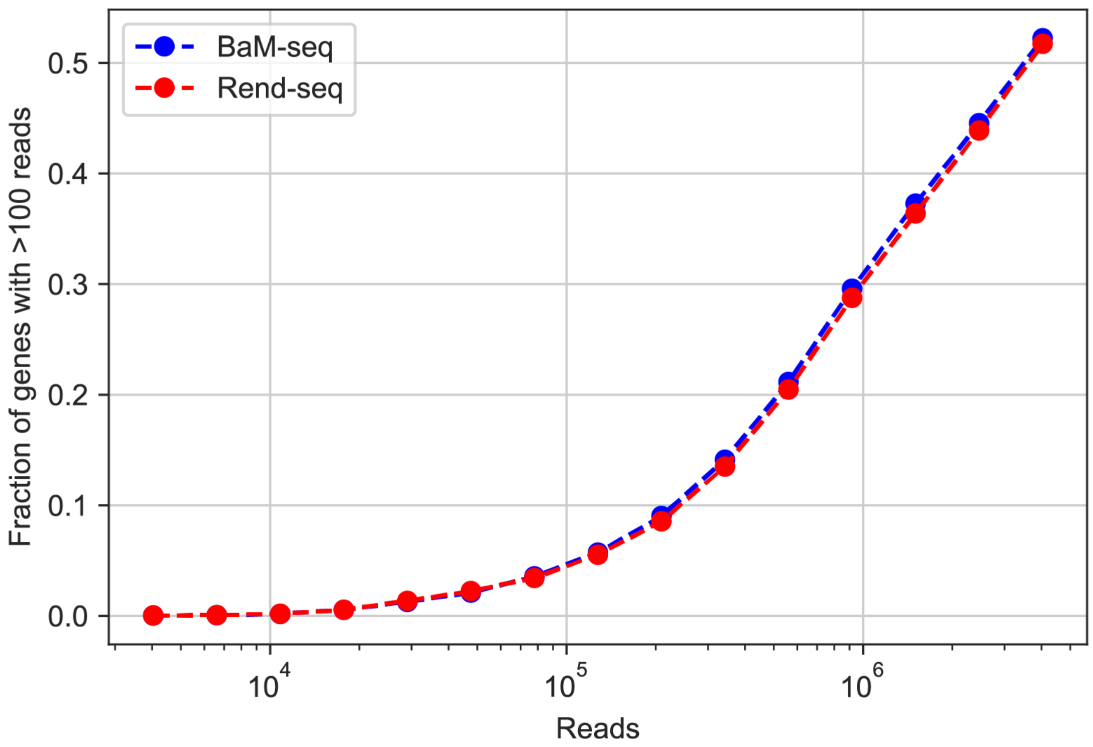

Figure S2. Comparison of BaM-seq and Rend-seq sensitivity. Read-level subsampling was performed on a representative BaM-seq and Rend-seq sample and the fraction of total genes with greater than 100 reads calculated for each subsampling.

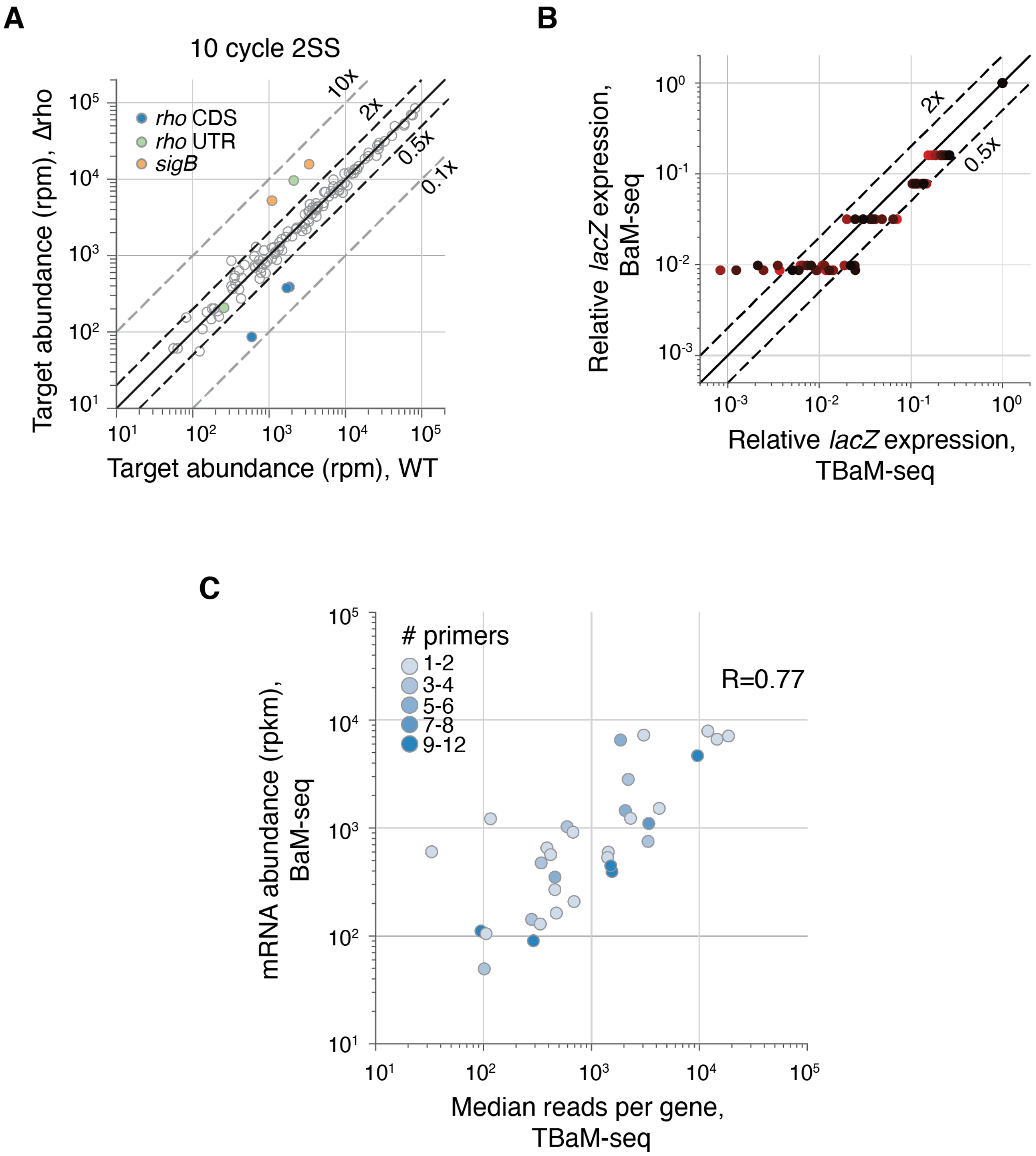

Figure S3. Additional validation of TBaM-seq workflow. (A) Reads per million primer-mapping reads across primers between WT and Δ*rho* samples, as in Fig. 4C, but with libraries generated with a second strand synthesis reaction with 10 cycles rather than 1. Primers targeting *rho* CDS and UTR, as well as those targeting *sigB* are highlighted. (B) *lacZ* expression relative to sample with highest *lacZ* induction. Expression for BaM-seq samples calculated as in Fig. 4D.

Expression for TBaM-seq samples is calculated as rpm for each *lacZ*-targeting primer and each primer plotted as a distinct color. (C) Comparison of relative gene expression for targeted genes (for pool 1 primers, as described by Fig. S3). Gene expression in BaM-seq calculated as rpkm. Gene expression in TBaM-seq calculated as median normalized reads across gene targeting primers.

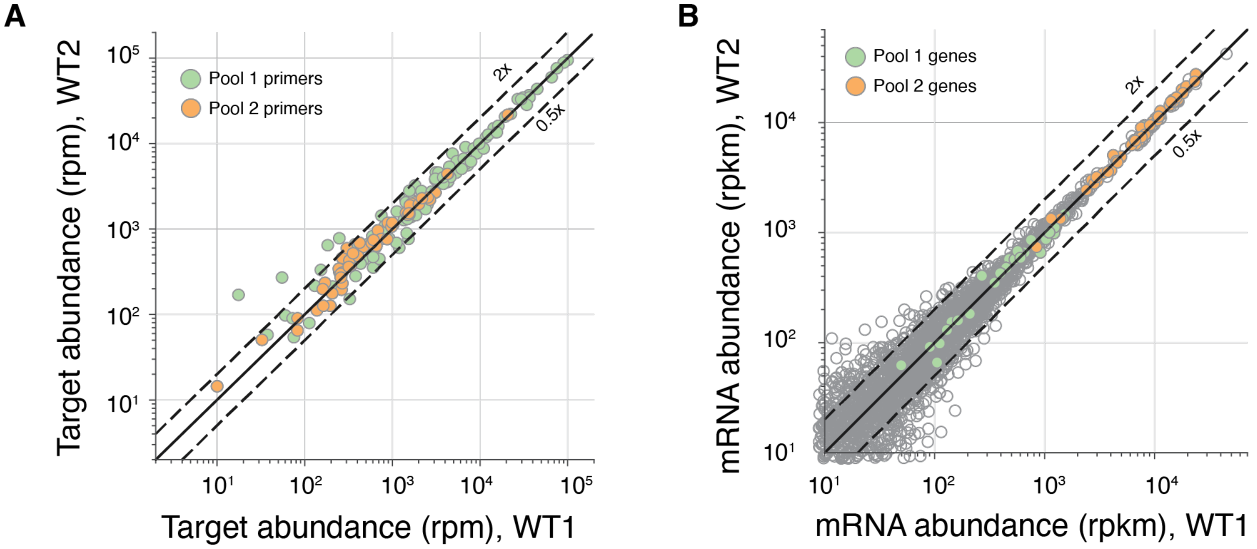

Figure S4. Redistribution of reads. (A) Two pools of primers, pool 1 and pool 2, were used to capture transcripts in two separate second-strand synthesis reactions. Following PCR, samples were re-pooled and sequenced. Normalized reads per primers in each pool are highlighted. (B) Relative expression across two BaM-seq samples. Genes targeted by pool 1 or pool 2 primers are highlighted.

Supplementary Data

Summary_mapped_reads.xlsx
Breakdown of specific and non-specific reads in TBaM-seq datasets.

TBaM-seq primers.xlsx
Gene specific sequences for TBaM-seq 2nd strand synthesis primers

Supplementary Methods

MULTIPLEX RNA-SEQ PROTOCOL

Input into this protocol is DNase treated, rRNA depleted RNA

Fragmentation

- Set up **250 ng RNA** in 10 µL ddH$_2$0 on a cold block
- Add 1 µL 10x Fragmentation reagent, briefly vortex to mix and spin down
- Incubate 95°C for 1 min 45 seconds & return to cold block
    - Best practice is to only perform up to 4 samples at one time to reduce fragmentation time variation
- Add 1.1 µL 10X Stop Solution
- Briefly mix & spin down
- Add 40 µL DEPC H$_2$0 & 100 µL Oligo Binding Buffer (Zymo)
- Vortex to mix & spin down
- Move to a fresh tube containing 400 µL 100% Ethanol & Vortex
- Transfer to Zymo Oligo Clean & Concentrator Column & spin 30s
- Decant flowthrough & add 750 µL Wash Buffer
- Spin 30s, decant, spin another 1 min
- Add 16 µL DEPC H$_2$0 to column, let stand 1 min, spin 30s

One Pot RT reaction [4 Hours]

- Combine in strip tube on cold block:
    - 16 µL RNA
    - **4 µL PNK Master Mix:**
        - 2 µL 10X PNK Buffer
        - 0.25 µL SUPERase*In
        - 1.25 µL DEPC H$_2$0
        - 0.5 µL PNK enzyme
- Incubate 37°C for 60 min, 75°C for 10 min
- Add **10 µL PolyA Master Mix:**
    - 3 µL 500 mM KCl
    - 3 µL 10 mM ATP
    - 2 µL 5X FS Buffer (From Superscript III enzyme)
    - 0.25 µL SUPERase*In
    - 1.25 µL DEPC H$_2$0
    - 0.5 µL E. Coli PolyA Polymerase (NEB M0276)
- Incubate 37°C for 30 min, 75°C for 10 min
- Add **1 µL 25 uM RT Barcoding Primer**
- Incubate 65°C for 5 min & return to cold block
- Add **Reverse Transcription Master Mix:**
    - 3 µL 0.1M DTT
    - 2 µL 10 mM dNTP mix
    - 2 µL 5X FS Buffer

    - 1.25 µL DEPC water
    - 0.25 µL SUPERase*In
    - 0.5 µL SS III RT Enzyme
- Incubate 50°C for 60 min, 75°C for 10 min
- **Pool all samples** into a single 1.7 mL tube & mix thoroughly
- Add 1/10 volume 1M NaOH & vortex briefly
- Incubate 95°C for 15 minutes

cDNA Size selection Gel [4 hours]
- Remove 180 µL pooled sample and add 90 µL 2X Urea loading buffer
- Heat samples for 2 min @ 75°C
- Load onto 8 lanes on 10% TBU gel
- Run gel at 200V for 1.5 to 2 hours
- Cut bands from >100 – 120 nt (see below for representative gel)
- Extract and precipitate*
- Dissolve pellet in 20 µL 10 mM Tris 8

3' end ligation [overnight]
- Add:
    - 10 µL Size-selected cDNA as substrate
    - 5 µL 100uM p214
    - 3 µL DEPC water
    - 5 µL 10X T4 DNA Ligase Buffer
    - 5 µL 5M Betaine
    - 20 µL PEG 8000
    - 2 µL T4 DNA Ligase
- Complete one reaction with 1 pmol oDJP208 as a ligation control
- Incubate at 16°C for 10 hours, 4°C overnight
- Heat to 75°C for 10 minutes to denature enzyme
- Clean up reaction with Zymo Oligo Clean & Concentrator column
- Elute in 10 µL 10 mM Tris 8
- Add 10 µL 2X Urea Loading dye
- Heat 95°C for 2 minutes
- Run out on a 10% TBU gel for 1 hour 45 minutes
    - Save one lane for oDJP208, oDJP209, control (oDJP208) reaction
- Cut band from ~135 – 155 (see below for representative gel)
- Extract and precipitate*
- Dissolve pellet in 20 µL 10 mM Tris 8

Library prep PCR [2 hours]

- Set up 5 PCR reactions per sample – 6, 8, 10, 12, 14 cycles
- Make master mix:
    - 5 µL Ligated DNA
    - 6 µL 10 uM oDP161

    - 6 μL 10 uM oDP128
    - 6 μL 10 mM dNTP mix
    - 24 μL 10X Q5 buffer
    - 60 μL water
    - 2 μL Q5 polymerase
- Aliquot 20 μL mix into 5 tubes, one for each cycle
- PCR Protocol
    - 98° - 30s
    - X cycles:
        - 98° - 10s
        - 60° - 10s
        - 72° - 7s
            - Pull tubes after elongation cycle completes!
    - 72° - 20s
- Add loading dye & run on 8% TBE gel at 180V for 55 minutes
- Cut PCR bands (see below for representative gel) – want to use lowest PCR cycle number that still provides enough product for sequencing in order to minimize PCR jackpot effects
- Extract and precipitate*
- Dissolved in 10 μL 10 mM Tris 8
- Send for sequencing!

Repeated Protocols, Buffers, Reagents

Precipitation Protocol:

1. To RNA or DNA add up to 300 total µL appropriate 10 mM Tris buffer (7 for RNA, 8 for DNA), 33 µL (1/10 volume) 3M Sodium Acetate pH 5.5, 2 µL Glycoblue reagent
2. Briefly vortex or mix by pipet & briefly spin down
3. Add 900 µL (3 volumes) 100% cold ethanol and briefly vortex
4. -80° freezer for at least 30 minutes
5. Spin in 4° centrifuge for 30 minutes
6. Decant ethanol and add 500 µL 70% cold ethanol
7. Spin for at least 5 more minutes
8. Decant and remove excess ethanol by pipet
9. Air dry for 5 minutes and then add appropriate water/Tris buffer

Gel extraction protocol

1. Pierce an 0.5 mL tube with an 18 gauge needle and put it inside a non-stick 2 mL screw cap tube.
2. Excise gel piece and place inside 0.5 mL tube.
3. Spin the nested tubes 3 min @ 20,000 xG to force the gel through the needle hole. Shake any residual gel from the small tube into the larger tube.
4. Add 500 µL DEPC water to gel pieces and incubate 10 minutes @ 70 C.
5. Vortex gel slurry >15 seconds and use cut p1000 tip to transfer gel mixture to Costar Spin-X column.
6. Centrifuge 3 min @ 20,000 xG to recover the elution mixture free of gel debris.
7. Transfer eluate to new non-stick tube.
8. Add 2 µL glycoblue, 45 µL 3M NaOAc for RNA (3M NaCl for DNA) to eluate.
9. Add 750 µL isopropanol and mix well, precipitate at least 30 minutes at -20 or -80 C.
10. Spin 30 minutes @ 20,000 xG @ 4 C to pellet nucleic acids.
11. Remove supernatant, wash pellet in 750 µL 80% ice-cold EtOH.
12. Spin >5 minutes, remove supernatant and air-dry.
13. Re-suspend in water or 10 mM Tris 7.

<u>Primers & Oligos & Enzymes</u>

Commercial Enzymes & Reagents

Enzyme	Supplier	Catalog Number
Fragmentation Reagent	ThermoFisher Scientific	AM8740
T4 Polynucleotide Kinase	NEB	M0201S
E. coli PolyA Polymerase	NEB	M0276L
Superscript III Reverse Transcriptase	ThermoFisher Scientific	18080093
T4 DNA Ligase	NEB	M0202M
Q5 DNA Polymerase	NEB	M0491L

General Reverse Transcription primer - CAAGCAGAAGACGGCATACGAGAT XXXXXX GTGACTGGAGTTCAGACGTGTGCTCTTCCGATCTTTTTTTTTTTTTTTTTTVNN

Reverse Transcription Primers

BC#	DJP Oligo#	Sequence	BC#	DJP Oligo#	Sequence	BC#	DJP Oligo#	Sequence
BC1	190	AAGTCC	BC20	244	CTACTA	BC39	1684	TCGTAA
BC2	191	ACTGCA	BC21	248	CTTGTT	BC40	1685	AACCCT
BC3	192	AGAACT	BC22	249	GACCAA	BC41	1686	TGGGAT
BC4	193	AGTCAC	BC23	1668	GATAGT	BC42	1687	GGGTTA
BC5	194	ATCATG	BC24	1669	GCAACA	BC43	1688	AGAGGA
BC6	195	CACGAT	BC25	1670	TTCGCA	BC44	1689	GAAGCT
BC7	196	CATGTC	BC26	1671	AGCTTT	BC45	1690	CTGCAT
BC8	197	CTGGTA	BC27	1672	ACAAGC	BC46	1691	TCCAGA
BC9	198	GAGCTT	BC28	1673	TATCGA	BC47	1692	ATCGGT
BC10	199	GATTCG	BC29	1674	CAAAGA	BC48	1693	GTATTC
BC11	200	GCTCAA	BC30	1675	TAGACA	BC49	1694	TCCGTT
BC12	201	GTCTGA	BC31	1676	CTAGAG	BC50	1695	CCAATT
BC13	202	TCACTG	BC32	1677	GCTATG	BC51	1696	CACTCA
BC14	203	TGCTAG	BC33	1678	AAGGAG	BC52	1697	CTTAGC
BC15	204	TGTTGC	BC34	1679	CCTTAG	BC53	1698	TACTGT
BC16	205	TTGAGT	BC35	1680	TCTACT	BC54	1699	CAATAC
BC17	241	ATGAAC	BC36	1681	ATACCC	BC55	1700	GGACAT
BC18	242	TATGCG	BC37	1682	TGAAAC	BC56	1701	AACGTA
BC19	243	GCGAAT	BC38	1683	GTAAGG	BC57	1702	TGGATG

cDNA Ligation oligos

DJP Oligo #	Use	Sequence
oDJP214	Ligation Oligo	AGATCGGAAGAGCGTCGTUCTGAUCTNNNN
oDJP208	Oligo for control reaction	CAAGCAGAAGACGGCATACGAGATGGACTTGTGACTGG AGTTCAGACGTGTGCTCTTCCGATCTTTTTTTTTTTTTTT TTTCGCGTTGCGGGTCGACTCCGTGTACAT
oDJP209	Final product control oligo	CAAGCAGAAGACGGCATACGAGATGGACTTGTGACTGG AGTTCAGACGTGTGCTCTTCCGATCTTTTTTTTTTTTTTT TTTCGCGTTGCGGGTCGACTCCGTGTACATAGATCGGAA GAGCGTCGTTCTGATCTNNNNNN

PCR Primers

DJP Oligo #	Alt. name	Sequence
oDJP121	oDP007	AATGATACGGCGACCACCGAGATCTACACTCTTTCCCTACA CGACGCTCTTCCGATCT
oDJP161	oDJP010	CAAGCAGAAGACGGCA

Sample gels

Post-RT gel

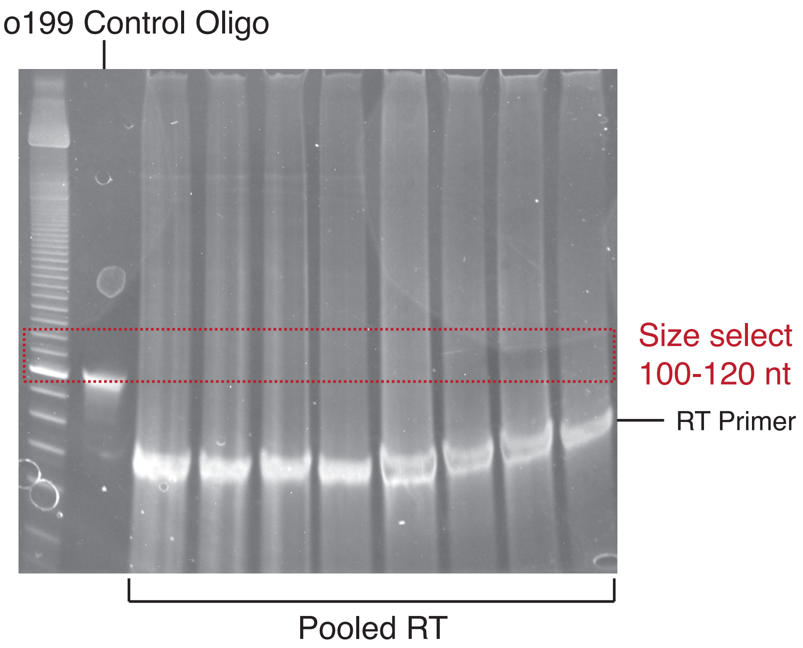

Post ligation gel

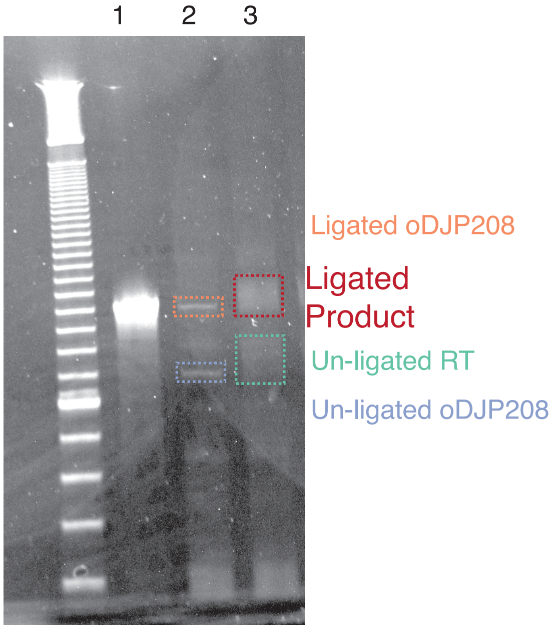

Final PCR

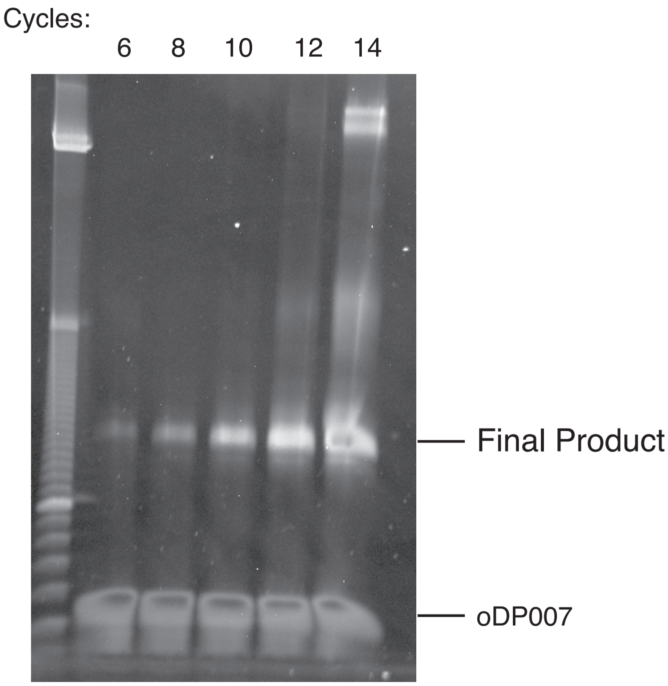

TARGETED RNA-SEQ PROTOCOL

Input into this protocol is barcoded, and pooled RT product generated as described above with the following differences:

- *rRNA depletion is not necessary; total DNase treated RNA samples can be used as input*
- *Run fragmentation reaction for 30s*
- *A minimum of 6 samples must be pooled to ensure enough material is present for second strand synthesis reaction*

Size Selection [4 hours]:

- Remove 240 µL pooled RT reaction product and add 120 µL 2X Urea loading buffer
- Heat samples for 2 min @ 70ºC
- Load onto 16 lanes over two 10% TBU gels
    - It is important to not run any control oligos on this gel, as these can contaminate the second strand synthesis reaction
- Run 1 hr. 45 min. @ 200V
- Cut bands from 115 – 135 nt
- Extract and Precipitate as described above;
- Dissolve pellet in 40µL 10 mM Tris 8

2nd Strand Synthesis:

- Combine:
    - 32 µL 5x Phu HF Buffer
    - 3.2 µL 10 mM dNTPs
    - x µL Primer Mix (add 1 pmol of each primer)
    - 4.8 µL DMSO
    - 1.6 µL Phu Polymerase
    - 40 µL RT rxn
    - H2O to 160µL
- Split reaction over 8 PCR tubes (20 µL each)
- Incubate:
    - 98ºC, 30s
    - 58ºC, 15s
    - 72ºC, 30s
    - 4ºC, ∞

Second strand synthesis primers contain a gene specific sequence, with the following common 5' end: CTTTCCCTACACGACGCTCTTCCGATCT

Size Selection [4 hours]:

- Add 10 µL 2X Urea Loading Buffer to each reaction.
- Heat 70ºC, 2 min
- Run over 8 lanes on 10% TBU gel, 1hr 45 min @ 200V

    - Do not run anything else on this gel (besides ladder)!
- Cut 145 – 165 nt
    - Cannot see material to cut on gel
- Extract and precipitate
- Dissolve pellet in 20μL Tris8

Library Prep PCR:

- Final PCR as above for multiplex RNA-seq with 16, 18, 20, 22, 24 cycles

Sample gels

Post second strand synthesis

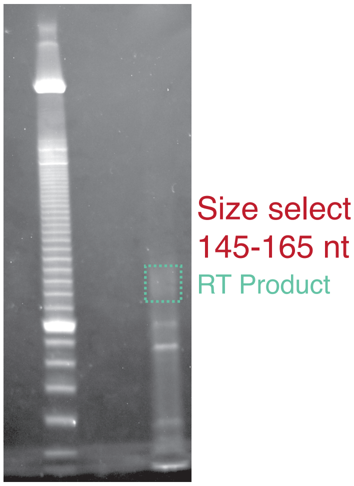

Final PCR

The desired product is the top band of a set of 3 bands. The smaller two bands are nonspecific product that are also seen when RT product rather than 2nd strand synthesis product is used as the input material for the PCR.

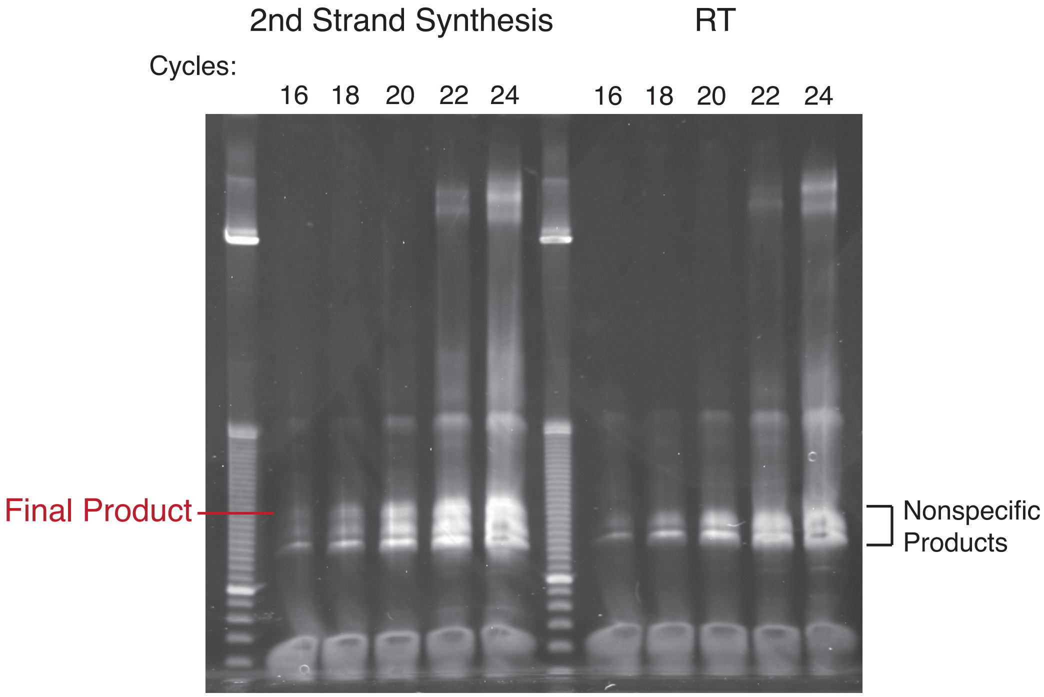

Supplement: lqad017_Supplemental_Files [file lqad017_supplemental_files.zip › 230112_supplementary_material.pdf]
